# Age-related differences in humerothoracic, scapulothoracic, and glenohumeral kinematics during elevation and rotation motions

**DOI:** 10.1016/j.jbiomech.2021.110266

**Published:** 2021-01-23

**Authors:** Christopher W. Kolz, Hema J. Sulkar, Klevis Aliaj, Robert Z. Tashjian, Peter N. Chalmers, Yuqing Qiu, Yue Zhang, K. Bo Foreman, Andrew E. Anderson, Heath B. Henninger

**Affiliations:** 1 – Department of Orthopaedics, University of Utah, Salt Lake City, UT; 2 – Department of Biomedical Engineering, University of Utah, Salt Lake City, UT; 3 – Department of Physical Therapy and Athletic Training, University of Utah, Salt Lake City, UT; 4– Department of Epidemiology, University of Utah, Salt Lake City, UT

**Keywords:** shoulder, age, rotation, abduction, kinematics

## Abstract

Age affects gross shoulder range of motion (ROM), but biomechanical changes over a lifetime are typically only characterized for the humerothoracic joint. Suitable age-related baselines for the scapulothoracic and glenohumeral contributions to humerothoracic motion are needed to advance understanding of shoulder injuries and pathology. Notably, biomechanical comparisons between younger or older populations may obscure detected differences in underlying shoulder motion. Herein, biplane fluoroscopy and skin-marker motion analysis quantified humerothoracic, scapulothoracic, and glenohumeral motion during 3 static poses (resting neutral, internal rotation to L4-L5, and internal rotation to maximum reach) and 2 dynamic activities (scapular plane abduction and external rotation in adduction). Orientations during static poses and rotations during active ROM were compared between subjects <35 years and >45 years of age (N=10 subjects per group). Numerous age-related kinematic differences were measured, ranging 5–25°, where variations in scapular orientation and motion were consistently observed. These disparities are on par with or exceed mean clinically important differences and standard error of measurement of clinical ROM, which indicates that high resolution techniques and appropriately matched controls are required to avoid confounding results of studies that investigate shoulder kinematics. Understanding these dissimilarities will help clinicians manage expectations and treatment protocols where indications and prevalence between age groups tend to differ. Where possible, it is advised to select age-matched control cohorts when studying the kinematics of shoulder injury, pathology, or surgical/physical therapy interventions to ensure clinically important differences are not overlooked.

## Introduction

Shoulder injuries and pathologies differentiate by age. Shoulder dislocation and acromioclavicular joint separations typically occur in patients 20–40 years of age (Chillemi et al., 2013; Zacchilli and Owens, 2010). Degenerative rotator cuff pathology is uncommon in patients under 50 (Lazarides et al., 2015; Minagawa et al., 2013), and glenohumeral (GH) osteoarthritis is uncommon in patients under 60 (Thomas et al., 2016). Shoulder arthroplasty, both anatomic (TSA) and reverse (rTSA), is not commonly performed in patients under 55. Data suggests that age is a significant factor related to functional outcomes and revision rate following rotator cuff repair and shoulder replacement surgery (Anakwenze et al., 2017; Lazarides et al., 2015). Clinical management in younger patients is met with higher expectations due to greater activity levels, frequent return to sport, and the need for more durable outcomes (Henn et al., 2011; Lazarides et al., 2015). Therefore, understanding the relationship between age and shoulder motion helps clinicians better manage expectations of functional range of motion (ROM) and ultimately patient satisfaction.

Age affects shoulder function (Barnes et al., 2001; Murgia et al., 2018). Humerothoracic (HT) ROM decreases with age for abduction and external rotation, but increases with age for internal rotation (Barnes et al., 2001). A stable scapula, capable of pain free range of motion in coordination with the humerus, is the basis for shoulder ROM (Paine and Voight, 2013), therefore abnormal scapular motion impairs shoulder function (Kibler et al., 2013). HT motion is the sum of GH and scapulothoracic (ST) contributions, which are each challenging to accurately measure since 3D movement of the scapula and humerus beneath soft tissue requires advanced imaging or invasive techniques to precisely quantify (Bourne et al., 2007; Tempelaere et al., 2016). Therefore, age-related scapular and glenohumeral contributions to shoulder motion remain understudied, yet quantification is necessary to better understand their effects on shoulder injury, pathology, or clinical interventions.

Recruitment of subjects with healthy shoulders over age 45 is challenging due to a higher prevalence of pathology and past injury (Tempelhof et al., 1999; Thomas et al., 2016). Thus, many studies have used healthy control populations under 35 years old for comparisons to clinical populations over 45 years of age (Bey et al., 2011; Hebert et al., 2002). Mismatched age groups may confound the conclusions of these studies, so it is important to establish the effect of age on ST and GH motion to determine if age-matched populations are necessary.

The purpose of this study was to compare healthy HT, ST, and GH kinematics in subjects <35 and >45 years of age. We hypothesized that the >45 population would exhibit (1) less HT ROM during scapular plane abduction and external rotation and (2) greater HT ROM during internal rotation, and (3) ST and GH kinematics would differ by age.

## Methods

### Participants

After obtaining informed consent (IRB 71782), ten healthy subjects <35 years of age and ten healthy subjects >45 years of age participated in a study of shoulder motion. These age groups were motivated by the relative distribution of shoulder injury and pathology described previously. Subjects recruited from the University of Utah were in good health (no preconditions on activity level), BMI ≤30, with equal distribution of sex per group. Subjects were screened for gross shoulder abnormalities and any history of shoulder surgery or pain. After pre-screening, anteroposterior and axillary radiographs were obtained to examine parameters like joint congruency and spacing. Magnetic resonance imaging followed standard shoulder protocols for our institution (e.g. axial PD FS; coronal T1, T2 FS; sagittal T1, T2 FS, oblique T2 FS; 320×320 or 384×384 matrix; 3.5/3.8 mm or 5.0/5.5 mm slices; no contrast; Magnetom Avanto_Fit 1.5T, Siemens, Malvern, PA). A fellowship-trained, board-certified orthopedic shoulder surgeon (PNC or RZT) reviewed radiographic and magnetic resonance imaging for signs of pathology. Subjects determined to have asymptomatic pathology (e.g. rotator cuff tears, osteoarthritis) were excluded from further study.”

### Activities

**Figure 1.**
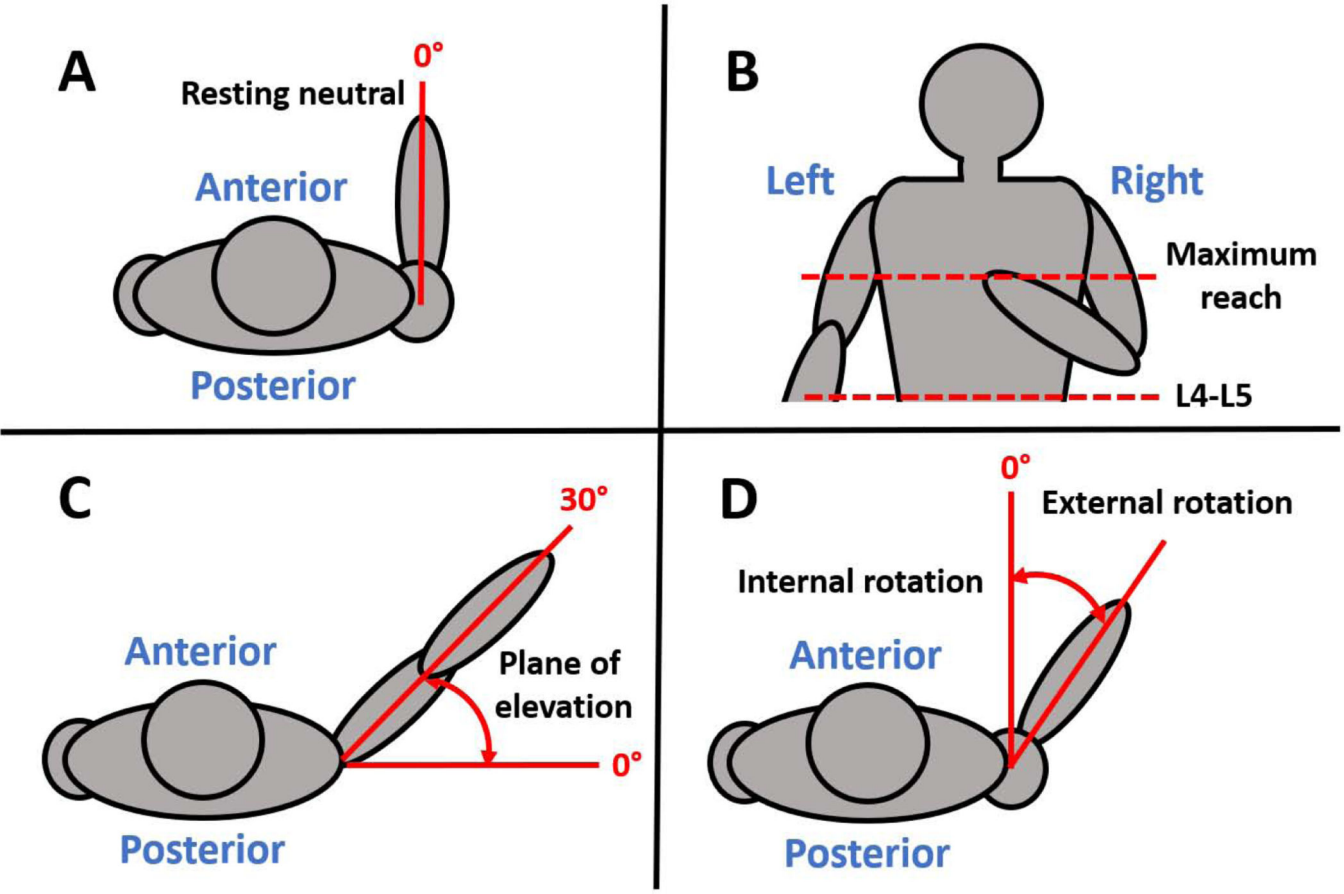
Schematic of resting neutral (static) (**A**), internal rotation (IR) in adduction (static) (**B**), scapular plane abduction (active) (**C**), and external rotation (ER) in adduction (active) (**D**). Resting neutral was defined with the arm at the subject’s side, the elbow flexed 90°, the forearm pointing anteriorly, and hand in a thumb up position. IR in adduction was performed to the L4-L5 spinal level and to maximum reach up the subject’s spine, with the arm behind the subject’s back and the palm open and facing posteriorly. Scapular plane abduction was defined as arm elevation with the subject’s elbow fully extended and the hand in a thumb up position, approximately 30° anterior to the coronal plane. ER in adduction was axial rotation of the arm at the subject’s side with the elbow flexed 90° and the hand in a thumb up position, beginning from maximal internal rotation with the forearm on the abdomen up to peak ER. Subjects performed scapular plane abduction at approximately 90° per second of elevation and external rotation in adduction at approximately 45° per second of rotation. Performance speeds were selected to be physiologically relevant and similar to previously reported rates (Bey et al., 2006; Phadke et al., 2011). Subjects were allowed to practice each motion prior to data capture until they felt comfortable with the motion and the timing.

Five poses/activities were studied: (1) static resting neutral, (2) static internal rotation (IR) in adduction to L4-L5, (3) static IR in adduction to maximum reach (i.e. thoracic levels), (4) dynamic scapular plane abduction, and (5) dynamic external rotation (ER) in adduction (Figure 1). These were selected since scapular plane abduction is the most commonly studied shoulder motion (Krishnan et al., 2019), and reaching behind the back (i.e. functional IR, a combination of extension and IR) plays a critical role in executing tasks of daily living (Langer et al., 2012). Neutral was used to quantify the resting pose of the scapula and humerus relative to the torso.

### Motion Capture and Analysis

A custom high-speed biplane fluoroscopy system (Radiological Imaging Services, Hamburg, PA) was used to image *in-vivo* shoulder kinematics at 100 Hz. A ten-camera motion analysis system (Vicon, Centennial, CO) was temporally and spatially synchronized to measure body motion outside the fluoroscope field of view. Subjects practiced the pose/motion prior to data capture to become familiar with the instructions. To reduce exposure to ionizing radiation, a trial was only repeated if the subject shifted out of the field of view or image quality was insufficient to allow markerless tracking. If multiple trials were captured, the last/best trial was analyzed. A computed tomography (CT) scan of each subject’s right shoulder followed standard imaging protocols for our institution (e.g. 130 kVp, 170 mAs with CareDose; 512×512 matrix; 1.0 mm slices (no overlap); no contrast; SOMATOM Definition AS, Siemens, Malvern, PA) capturing the entire scapula and humerus.. The scapula and humerus were semi-automatically segmented (Mimics, Materialise, Plymouth, MI) to generate 3D reconstructions.

**Figure 2.**
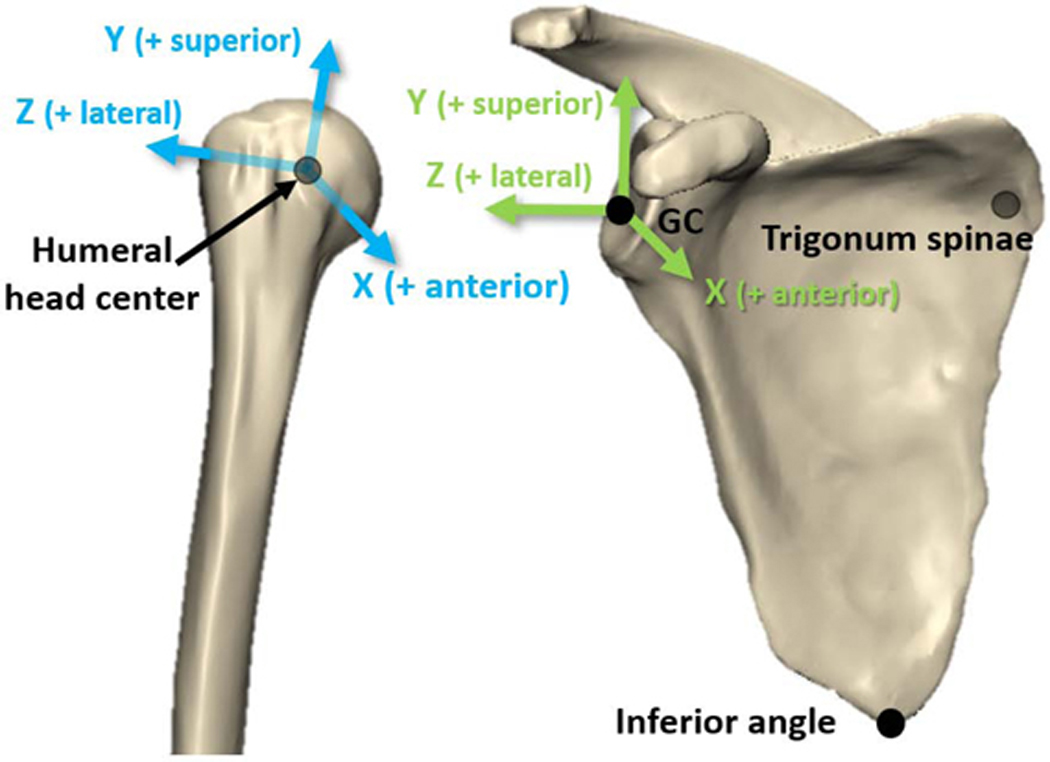
Local coordinate systems for the humerus and scapula. Humerus coordinate system: The origin was located at the centroid of a sphere fit to the articular surface of the humeral head. The vector from the midpoint of the elbow epicondyles to the humeral head center was defined as the humeral Y-axis (+superior). The normal to the plane defined by the humeral head center and the elbow epicondyles was the humeral X-axis (+anterior). The cross product of the humeral X- and Y-axes defined the humeral Z-axis (+lateral) (Wu et al., 2005). Scapula coordinate system: The origin was located at the glenoid center, defined by a circular fit of the inferior glenoid rim (De Wilde et al., 2010; Verstraeten et al., 2018). The vector from the trigonum spinae scapulae to the glenoid center was defined as the scapular Z-axis (+lateral). The normal to the plane defined by the glenoid center, inferior angle, and trigonum spinae scapulae was the scapular X-axis (+anterior). The cross product of the scapular Z- and X-axes defined the scapular Y-axis (+superior). Torso coordinate system (not shown): The origin was located coincident with the marker defining the incisura jugularis. The vector from the midpoint of the xiphoid process and T8 spinous process (obtained via anthropometric calculations) to the midpoint of the incisura jugularis and C7 spinous process defined the torso Y-axis (+superior). The normal to the plane defined by those four points was the torso Z-axis (+lateral). The cross product of the torso Z- and X-axes defined the torso X-axis (+anterior) (Wu et al., 2005). HT and GH rotations (*X-Z’-Y”*): Elevation (*X*), plane of elevation (*Z’*), and finally internal/external rotation (*Y”*). ST rotations (*Y-X’-Z”*): Protraction (*Y*), medial/lateral rotation (*X’*), and finally posterior tilt (*Z”*). Sign conventions for rotations followed the right-hand rule about the positive axes, but for more intuitive visual representation some were reversed from their coordinate system definitions (e.g. IR-/ER+, Depression-/Elevation+, and Medial Rotation-/Lateral Rotation+) as defined in the respective figure legends. This is a noted discrepancy between ISB and the International Shoulder Group definitions of elevation (Wu et al., 2005).

Model-based markerless tracking was used to semi-automatically align digitally reconstructed radiographs of the humerus and scapula with the corresponding fluoroscope images (Bey et al., 2006) (average Euclidian bias of 0.32±0.08 mm (pelvis) and 0.30±0.06 mm (femur) using the same system and software (Kapron et al., 2014)). Spatial and temporal calibration between marker and markerless systems allowed the torso (skin markers) to serve as the reference coordinate system to control for changes in posture and body habitus. Rotation matrices for HT, ST, and GH motions were calculated for each time point using subject-specific coordinate systems for the humerus, scapula, and torso (Figure 2). Each HT and GH rotation matrix was decomposed using the *X-Z’-Y”* sequence due to known issues numerically representing the underlying motion using the *Y-X’-Y”* sequence (Phadke et al., 2011). ST rotation matrices were decomposed using the *Y-X’-Z”* sequence (Wu et al., 2005). The glenoid center was used as the scapular origin (Kolz et al., 2020).

Scapulohumeral rhythm (SHR) was calculated two ways. First, as SHR=ΔGH/ΔST, where the change was calculated relative to the joint position at the start of the motion. Second, as SHR=δGH/δST, which was the instantaneous change relative to the prior increment of HT elevation. Since substantial reorientation can occur during dynamic motion, ROM was calculated as (max-min) across the motion, not (start-end). Therefore, ROM at times exceeded the difference between start and end orientations.

### Statistical Analysis

*A priori* power analyses using two independent means were performed using G*Power (v 3.1.9.4, University of Düsseldorf, Germany). Mean differences were conservatively estimated between ages for abduction (10–15°) and ER (7.5°) using previously recorded changes in ROM (Barnes et al., 2001). Standard deviations were estimated as 7.5–10° (Barnes et al., 2001; Schwartz et al., 2014). To achieve 0.8 power a sample size of 9 subjects per group was required.

All demographics and static poses, including the start and end positions from dynamic motions, were compared between age groups using two tailed independent t-tests assuming equal variance. For comparisons between static poses within an age group (e.g. rest versus maximal IR), paired t-tests with Holm-Bonferroni correction for multiple comparisons were used. Dynamic rotations were interpolated at 0.1° increments and Statistical Parametric Mapping (SPM) was used to compare between age groups. SPM uses a time-domain dependent analysis with the advantage that it enables temporal comparisons to compute cluster-based p-values (Bangerter et al., 2019). A previous implementation of SPM using independent t-tests was adapted for our analysis (1d version 0.4, www.spm1d.org) (Pataky et al., 2015). Statistical significance (P≤0.050) and marginal significance (0.050<P≤0.100) used to detect potential regions of difference for consideration in future shoulder kinematics studies.

## Results

### Demographics

Ten healthy subjects <35 years of age (5M/5F; 26±3 yrs; 171.4±11.1 cm; 60.9±13.1 kg; 20.6±3.4 kg/m^2^) and ten healthy subjects >45 years of age (5M/5F; 58±7 yrs; 173.1±6.9 cm; 79.0±13.9 kg; 26.2±3.4 kg/m^2^) were recruited. Weight and BMI for individuals over 45 years were greater compared to the group under 35 years of age (P≤0.008). No differences were detected for height between the two groups (P=0.687). All subjects were right-hand dominant, and all subjects that met screening and inclusion criteria completed the entire study protocol. One subject (<35) was excluded from the analysis due to an incomplete marker set during post-processing calibration, preventing calculation of poses and orientations relative to the torso coordinate system. One subject (>45) was excluded from scapular plane abduction and a different subject (>45) was excluded from IR/ER in adduction due to poor image quality that prevented tracking of those trials.

### Static Poses

**Figure 3.**
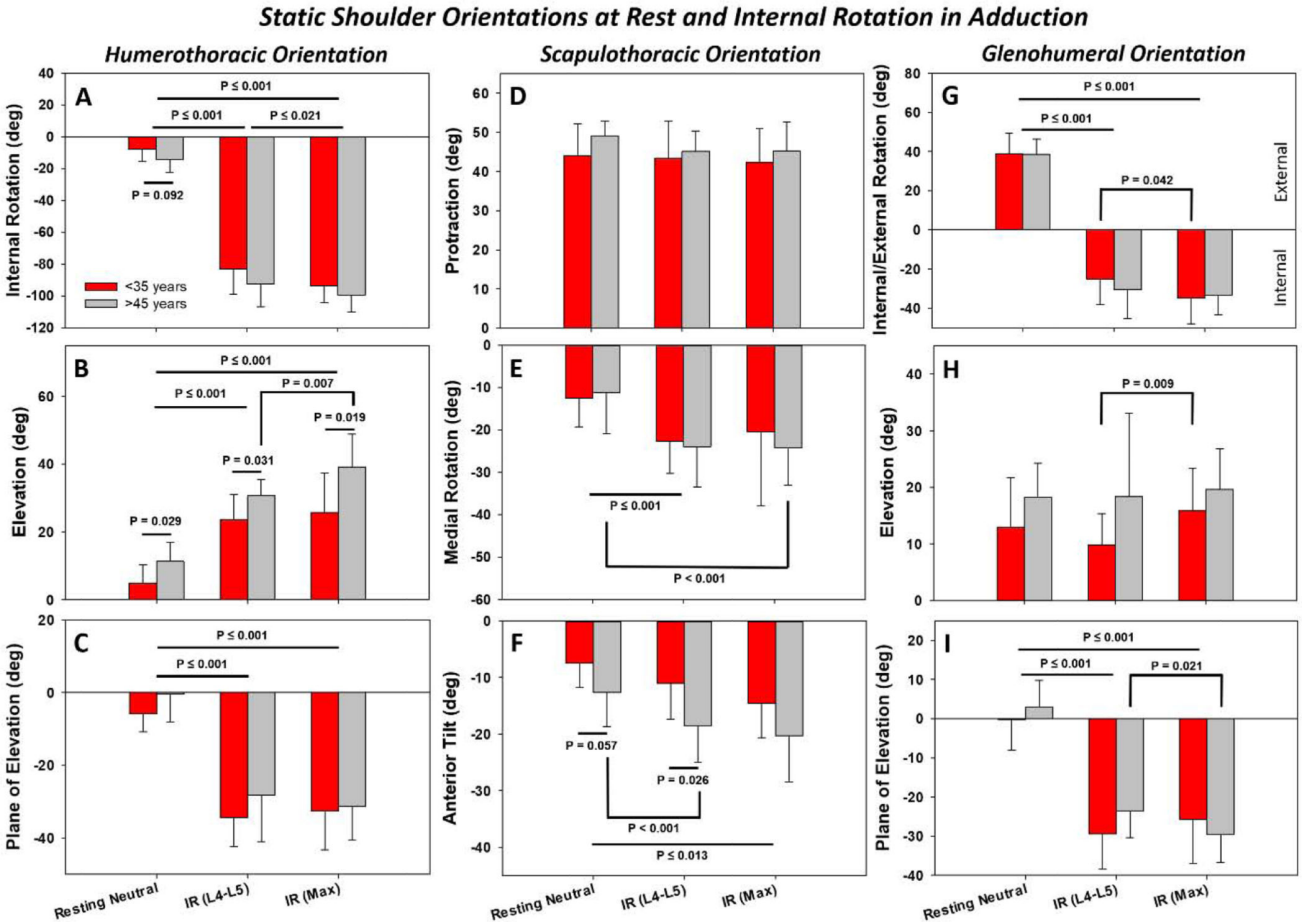
Humerothoracic (**A-C**), scapulothoracic (**D-F**), and glenohumeral (**G-I**) orientations during static resting neutral, internal rotation (IR) (L4-L5), and IR (max) positions, grouped by age. Most differences in joint orientations were detected between the resting neutral and IR poses (A, B, C, E, F, G, I) but some differences between poses only arose within one age group (e.g. <35 G, H; >45 B, E, F, I). Age yielded clear differences in humerothoracic elevation (B), with marginal significance at rest for humerothoracic IR/ER (A). Data are presented relative to the torso coordinate system as mean±SD. Note that for more intuitive visual representation some sign conventions were reversed from their coordinate system definitions (e.g. IR-/ER+, Depression-/Elevation+, and Medial Rotation-/Lateral Rotation+).

For both age groups, IR poses had 20° or more HT internal rotation, elevation, and plane of elevation than resting neutral orientations (P≤0.001) (Figure 3 A-C). Subjects over 45 years of age experienced approximately 10° greater HT elevation during resting neutral, IR to L4-L5, and IR to maximum reach (P≤0.031) (Figure 3B). There were no differences by age for plane of elevation, but marginal significance was detected between ages in resting internal rotation (~10°, P=0.092) (Figure 3A).

For the ST joint, no differences were detected in scapular protraction by pose or age (Figure 3D). To the contrary, the scapula experienced approximately 10° more medial rotation (P≤0.001) (Figure 3E) and approximately 5° anterior tilt (P≤0.013) (Figure 3F) for IR poses compared to neutral for both age groups, with the exception of maximum IR in the <35 years group. While subjects >45 years tended toward higher mean protraction, tilt, and rotation than younger subjects in 8/9 cases, these differences only reached statistical significance for tilt (P≤0.057) (Figure 3F).

For the GH joint, internal/external rotation (Figure 3G) and plane of elevation (Figure 3I) inverted for IR poses compared to resting neutral. The L4-L5 and maximal IR poses differed for only 3/6 comparisons within an age group (P≤0.042) (Figure 3G-I). GH orientations did not differ by age. Individuals >45 years tended toward higher elevation (Figure 3H) but internal/external rotation and plane of elevation showed no consistent trends by age.

### Dynamic Activities

Total ROM differed statistically for HT and GH IR/ER during scapular plane elevation, where the >45 years group achieved mean 22° more HT rotation and 13° more GH rotation than the <35 years group (P≤0.029) (Table 1). Since the HT and GH rotation end orientations did not differ by age during scapular plane abduction, the change in ROM arose from differences in starting rotation of the arm (P≤0.034). No other variations in ROM were detected, but 11/36 start and end orientations demonstrated at least marginal statistical significance (P≤0.089). The ST joint was responsible for 5/11 statistical differences detected, namely in scapular tilt and protraction. The HT and GH elevation and plane of elevation made up the remainder of differences in start and end positions.

**Figure 4.**
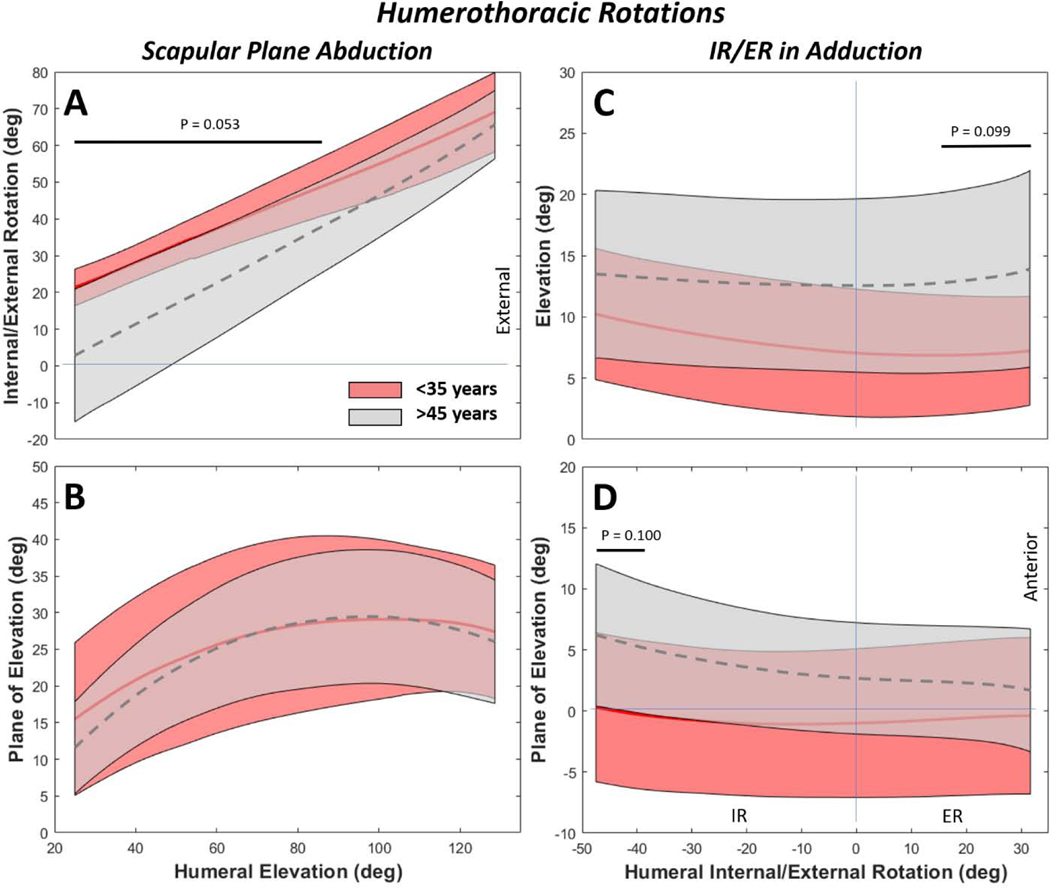
Humerothoracic rotations for scapular plane abduction (**A, B**) and internal/external rotation (IR/ER) in adduction (**C, D**). By age, statistical differences on the order of 10–20° were detected in IR/ER during scapular abduction (A), and approximately 5° for elevation and plane of elevation during IR/ER motions (C, D). Elevation during scapular plane abduction and IR/ER during ER in adduction are not shown because these act as the independent variables for the corresponding dynamic motions, resulting in a slope of 1, with no variance. Solid and dashed curves represent the mean and shaded regions represent the standard deviation of the respective populations. Note that for more intuitive visual representation some sign conventions were reversed from their coordinate system definitions (e.g. IR-/ER+, Depression-/Elevation+).

**Figure 5.**
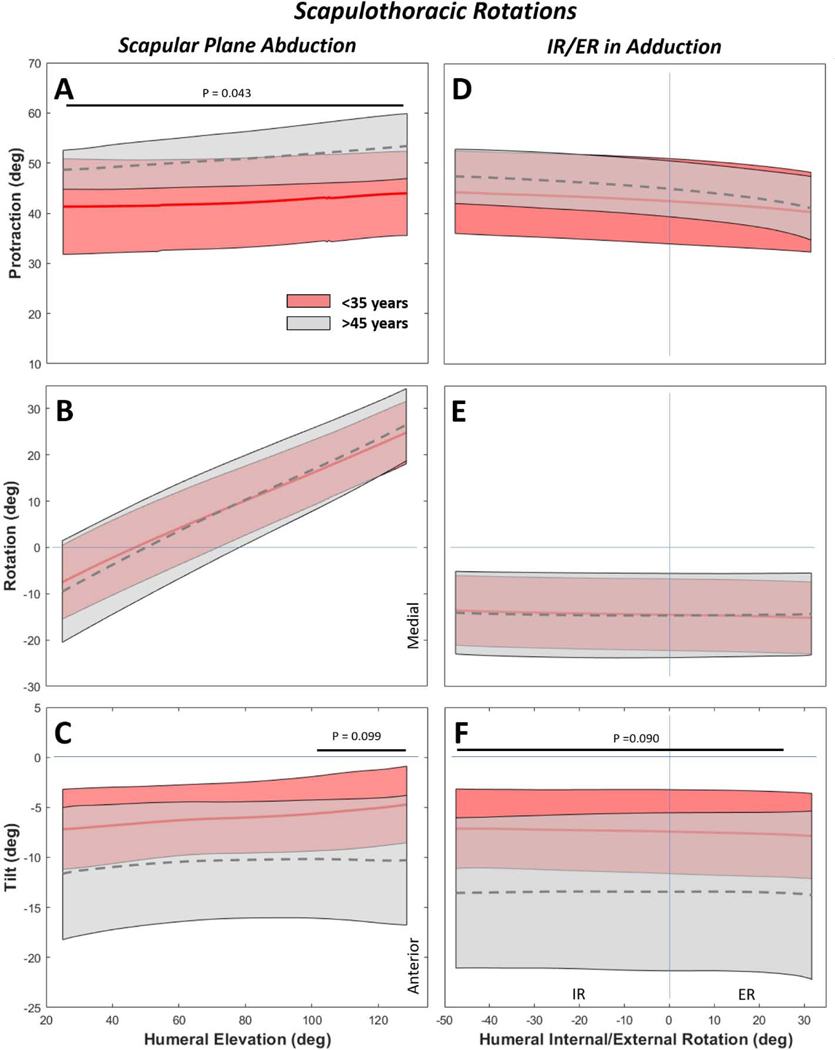
Scapulothoracic rotations for scapular plane abduction (A-C) and internal/external rotation (IR/ER) in adduction (D-F). By age, statistical differences of approximately 10° were detected in protraction (A) and 5° in tilt (C) during scapular abduction. During IR/ER in adduction the scapulothoracic rotation was nearly identical between age groups (E), but there were differences of approximately 7° detected for scapula tilt across the range of motion (F).Solid and dashed curves represent the mean and shaded regions represent the standard deviation of the respective populations. Note that for more intuitive visual representation some sign conventions were reversed from their coordinate system definitions (e.g. Medial Rotation-/Lateral Rotation+).

**Figure 6.**
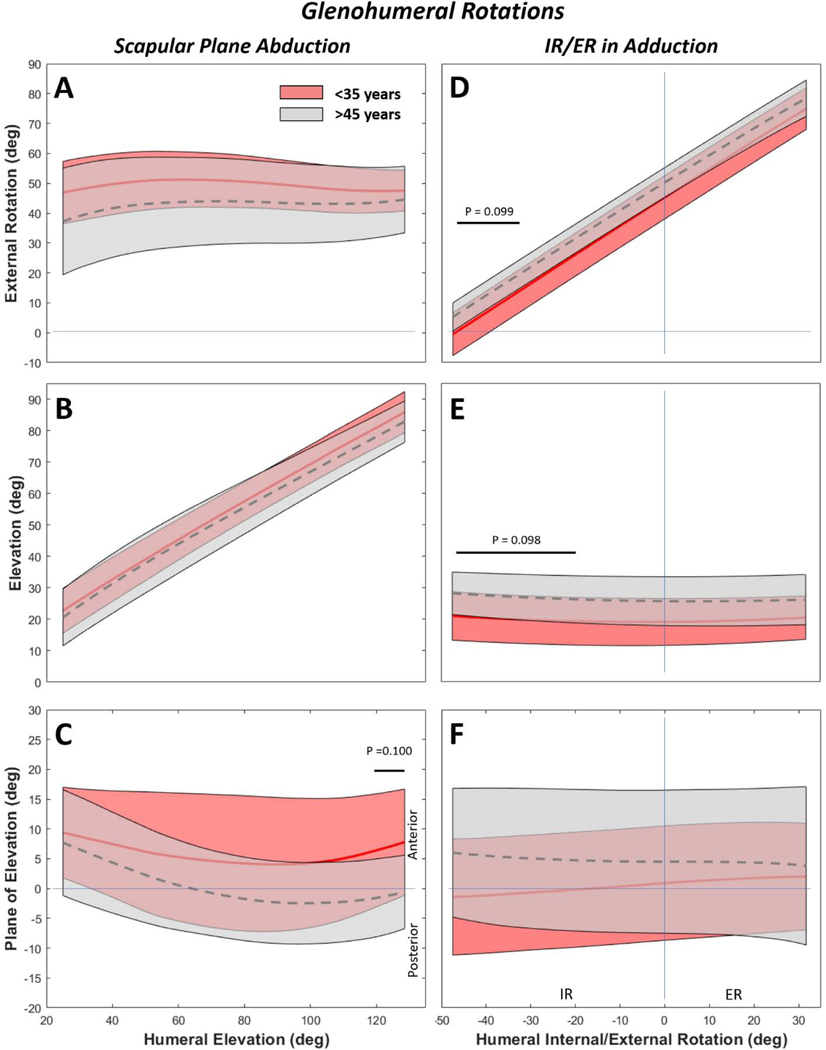
Glenohumeral rotations for scapular plane abduction (A-C) and internal/external rotation (IR/ER) in adduction (D-F). By age, statistical differences of approximately 10° were only detected in plane of elevation during scapular plane abduction (C). To the contrary, differences of 7–10° were detected for external rotation (D) and elevation (E) during IR/ER motions, with differences arising during the internal rotation component of the motion. Solid and dashed curves represent the mean and shaded regions represent the standard deviation of the respective populations. Note that for more intuitive visual representation some sign conventions were reversed from their coordinate system definitions (e.g. IR-/ER+, Depression-/Elevation+).

**Figure 7.**
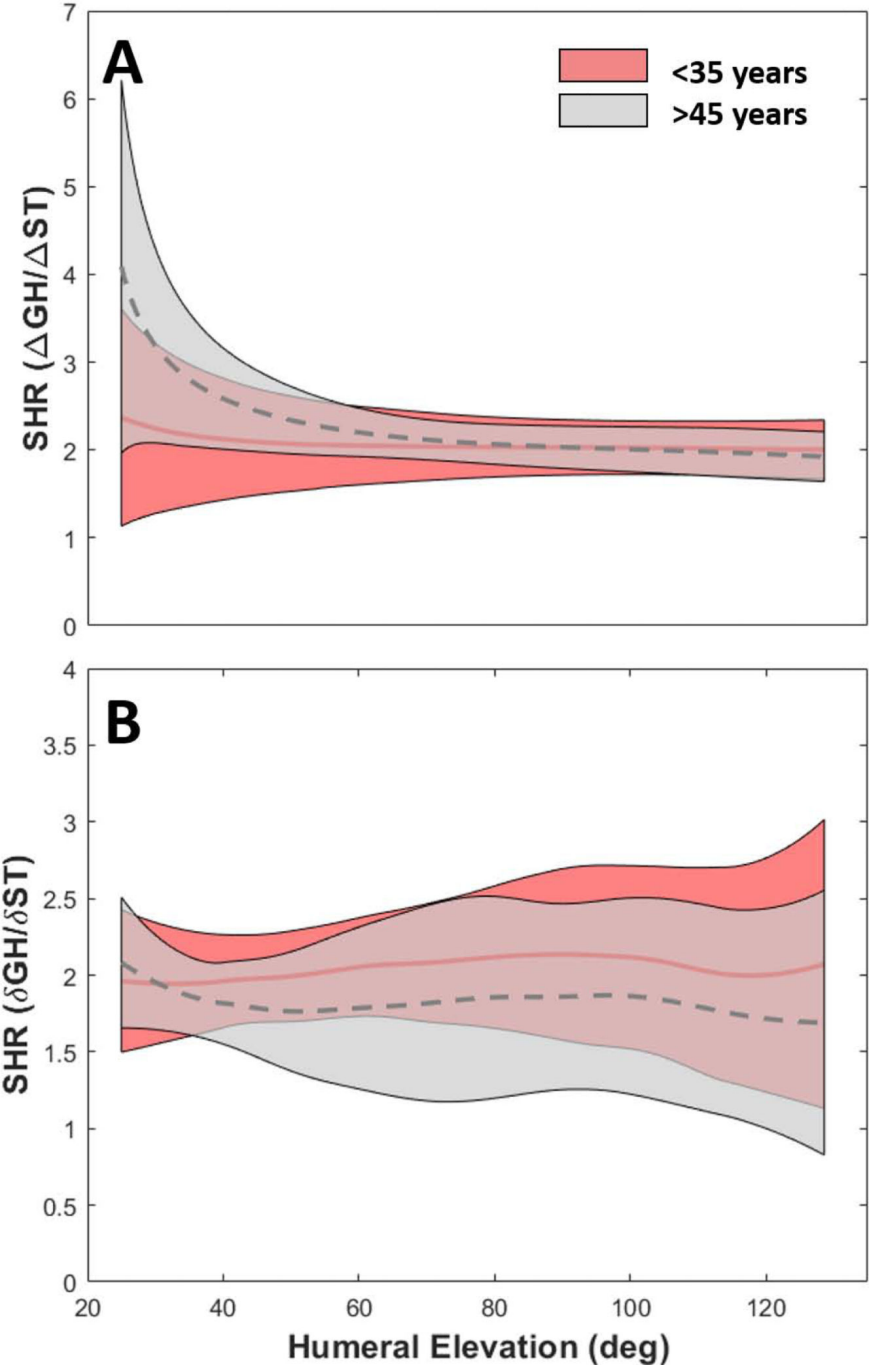
Scapulohumeral rhythm (SHR) during scapular plane abduction was calculated as the ratio of GH elevation to ST lateral rotation. **(A)** Here the SHR (Δ) represents the change relative to the initial position of the subject. Early in the elevation the SHR was dominated by glenohumeral humeral motion relative to scapulothoracic, resulting in SHR upward of 6 in some subjects, but stabilized around a 2:1 ratio above approximately 50° of humeral elevation. **(B)** Here the SHR (δ) represents the instantaneous change relative to the prior increment of elevation. A 2:1 ratio was observed across the entire range of humeral elevation. No statistical differences were detected between sexes.

Dynamic motions were also evaluated across the HT ROM achievable by all subjects: 25° to 125° for abduction and −45° to 35° for IR/ER (Figure 4–7).During HT scapular plane abduction, both groups exhibited an increase in external rotation with humeral elevation, but the <35-year group was up to 20° higher than the >45 group across much of the ROM (Figure 4A). No differences were observed in plane of elevation between age groups in scapular plane abduction (Figure 4B). During external rotation in adduction, the >45-year group demonstrated approximately 5° more humeral elevation and 5° more anterior plane of elevation than the <35 group, though they only statistically differed over a fraction of the ROM (Figure 4C,D).

The >45-year group consistently had 10° more scapular protraction than the <35-year group during scapular plane abduction (Figure 5A). While the groups did not differ in medial/lateral ST rotation (Figure 5B), statistical differences arose for scapular anterior tilt, where the >45-year group was more anteriorly tilted by approximately 5° (Figure 5C). External rotation in adduction did not yield any qualitative or quantitative differences in ST protraction or rotation (Figure 5D,E), but the >45-year group was approximately 7° more anteriorly tilted across the ROM (Figure 5F).

Interestingly, the <35-year group tended toward higher mean GH rotations during abduction (Figure 6A-C), but this relationship inverted during external rotation in adduction (Figure 6D-F). Scapular plane abduction yielded no statistical differences in GH external rotation or elevation (Figure 6A,B), but up to 10° difference between groups for plane of elevation near terminal ROM (Figure 6C). The >45-year group had more external rotation and elevation, primarily during the internal rotation component of the IR/ER activity (Figure 6D,E). No differences were detected in plane of elevation during IR/ER in adduction (Figure 6F).

The HT plane of elevation was achieved as instructed, with a mean near 30° at 90° elevation, which was the instructed position (Figure 4B). At 90° HT elevation the GH plane of elevation showed means <5° but spanned over 30° (Figure 6C), demonstrating the underlying variance in ST and GH relationships relative to HT alignment.

There were no statistical differences in SHR (Figure 7), which was dependent on the calculation. When referenced to initial shoulder orientation, subjects peaked near 6:1 then rapidly decreased, stabilizing near 2:1 (Figure 7A). As an incremental change in GH and ST motion, the SHR was consistently near 2:1 (Figure 7B).

## Discussion

The objective of this study was to quantify age-related differences in HT, ST, and GH kinematics to determine if mismatched selection of control populations could confound studies of shoulder motion. To meet this objective, the study measured shoulders in control subjects <35 and >45 years of age during three static poses and two dynamic motions using skin marker and biplane fluoroscopy motion capture systems. We hypothesized that the >45 population would exhibit (1) less HT ROM during scapular plane abduction and external rotation and (2) greater HT ROM during internal rotation, and (3) ST and GH kinematics would differ by age. Our first two hypotheses were unsupported, since only a few gross differences in HT ROM were detected, and none in the primary HT motions of scapular abduction and IR/ER in adduction. Our third hypothesis was generally supported since numerous statistical differences were detected in ST and GH kinematics by age. Scapulothoracic motion differed between groups in the range of 5–22° which is well above the accuracy of the biplane fluoroscopy system (Kapron et al., 2014), indicating they present as a function of age. Since nearly half of all measures demonstrated statistical differences between age groups, it is recommended that future studies of shoulder pathology and injury consider age-matching control and patient populations. With more laboratories using highly accurate motion analysis techniques like biplane fluoroscopy to examine shoulder motion, differences that were obscured using lower resolution techniques can no longer be overlooked.

Mean clinically important difference (MCID) defines the smallest difference of an intervention that a patient perceives as beneficial (Jaeschke et al., 1989). No MCIDs for ROM after rotator cuff repair exist, so a prior cuff repair study (Nazari et al., 2019) used values from shoulder arthroplasty where active abduction and external rotation following shoulder replacement have MCIDs of 7° and 3°, respectively (Simovitch et al., 2018). These were likely measured using a clinical goniometer, which exhibits standard errors of measurement of 5–8° (Muir et al., 2010), but is incapable of capturing accurate 3D shoulder motion. Mean differences in static poses and dynamic kinematics ranged from 5–22° (Table 1, Figures 3–6), which exceeds these MCIDs, including goniometer measurement error. Inappropriately aged control populations could inhibit the detection of clinically important differences or yield false positives.

Resting neutral orientations differed from IR behind the back for all but ST protraction (Figure 3). Differences were anticipated given the disparity in the poses, but scapular rotation and tilt, and GH rotation and plane of elevation, are the primary drivers for achieving IR positions. Lost IR due to pathology, injury, or surgical repair (Jain et al., 2013; Wirth et al., 2016) should focus on these variants to identify the source of the deficiency and potential approaches to mitigate the loss. Interestingly, age related differences of 5–10° only manifested in HT elevation and ST tilt. Perhaps higher BMI in the >45 group and consequent torso and arm girth required the humerus to deviate from the torso by a larger elevation angle, and decreased strength of the scapular stabilizing muscles allowed more anterior tilt. The origins of these differences should be studied to determine their effect on age-related shoulder kinematics.

Total ROM alone is likely not an adequate measure of *in vivo* shoulder function since it cannot readily differentiate the nuances of underlying ST and GH motion. Where prior studies noted differences by age (Barnes et al., 2001; Murray et al., 1985), our first two hypotheses did not support those observations. Only a few gross differences in HT ROM were detected, with none in the primary HT motions of scapular abduction and IR/ER (Table 1). This inconsistency may have arisen from measuring bone motion using biplane fluoroscopy versus lower resolution methods measuring body segments, that we expressed motions relative to a local torso coordinate system rather than global posture, or simply a lack of differences between groups. Regardless, most statistical differences arose in ST and GH joints which combine to generate HT motion. Here there were clear differences in ST and GH motion by age, on the order of 5–22°, for ST protraction and tilt (Figure 5) and GH rotation and elevation (Figure 6). In addition, the ST and GH poses differed more often than gross HT poses or ROM (Table 1). As clinical care and research focus more on deficiencies of ST motion and their effects on shoulder function, it will be critical to age match control cohorts so as to capture the inherent differences specific to ST and GH function.

Our SHR agrees with the generalized 2:1 ratio beyond ~50° HT elevation but is dependent on the calculation. As changes relative to the initial joint position (Δ, Figure 7A) the SHR near 6:1 illustrates the large contribution of GH versus ST motion in the early phase of abduction. When taken incrementally (δ, Figure 7B), both groups consistently achieved the 2:1 ratio across the ROM. While early SHR decay has been reported previously (Chung et al., 2019; Robert-Lachaine et al., 2016; Scibek and Carcia, 2012), others report nearly constant SHR (Inman et al., 1996; Ludewig et al., 2009; Matsuki et al., 2011). These seemingly contradictory reports are clearly affected by the calculation, but may also be affected by the data capture. The ST and GH measurements are often obscured by soft tissue artefact, which is avoided when using radiographic techniques like ours. Consistency in reporting is needed to reliably study pathology or procedures like rTSA. SHR decreases to ~1:1 after rTSA surgery, illustrating the increased demands on ST motion (de Toledo et al., 2012; Kwon et al., 2012; Walker et al., 2015). Given that rTSA patients are predominantly >55 years of age, along with our kinematics findings, comparisons to appropriately age matched cohorts are advised. Of note, SHR was essentially 2D, using only single axes of rotation for calculation of the proportion of GH to ST motion. Future work will examine if 3D SHR may overcome issues with identifying sensitive out of plane differences in motion (Lee et al., 2020).

Sex can also affect shoulder biomechanics (Murgia et al., 2018; Nagamatsu et al., 2015). For completeness, we performed a secondary analysis by sex (see Supplement). The ten female (42±18 yrs; 165.5±4.0 cm; 61.4±12.8 kg; 22.3±4.3 kg/m^2^) and ten male subjects (42±18 yrs; 179.1±7.4 cm; 78.5±14.8 kg; 24.5±4.4 kg/m^2^) were re-pooled, and height and weight differed (P≤0.013). Static poses yielded some differences by sex, but inconsistently between poses (Figure S1). Sex did not affect ROM, and only a few statistical differences were detected for start and end positions, primarily between GH elevation during scapular plane abduction (Table S1). This appeared as a gross offset in start and end positions, since GH elevation ROM was very similar. Sex had only a minor effect on shoulder kinematics during motion. No HT or ST differences were detected by sex (Figure S2, S3). The GH elevation differed by 5–10° across scapular plane abduction (Figure S4B) and GH ER differed by a similar magnitude during IR/ER in adduction (Figure S4D). The trends in SHR were similar to the analysis by age, where again no significant differences were detected by sex (Figure S5).

This study is not without limitations. Although we captured two disparate age groups, there is likely a gradient of change that occurs over a human lifetime and as a function of changing body habitus. Though in good agreement with prior high resolution 3D data for HT, ST and GH shoulder motion (Ludewig et al., 2009), our data should not be considered definitive in magnitude, but rather evidence to support the need for matching of control cohorts where possible. It is recognized that substantial challenges exist in identifying healthy shoulders since prevalence of both full thickness rotator cuff tears and GH arthritis each individually exceed 30% in older individuals (Minagawa et al., 2013; Thomas et al., 2016). Second, variations in study population size may affect results. The present sample size was small compared to the 280 subjects who participated in (Barnes et al., 2001). However, that study was only able to investigate HT angles using a goniometer, which is prone to measurement error. The use of biplane fluoroscopy provided highly accurate kinematic data, but this technique is time- and resource-intensive and exposes individuals to ionizing radiation, limiting the number of participants feasible for analysis. This may have decreased the number of statistical differences detected, but our power analysis supported N=9 subjects per group to measure conservative effect sizes. The fact that numerous differences were detected herein, with deviations between age groups up to 22°, is sufficient evidence to warrant cohort matching for analyses of shoulder function. Prior studies report skin-marker motion analysis errors for the shoulder that exceed 10° (Cereatti et al., 2015), indicating that high resolution techniques may be required to discern clinically important differences below the levels perceptible using lower resolution measurements. This is especially important if ST and GH motion is critical to the research question under study. Finally, right shoulders were tested without external applied loads, but side, relative effort, and strength affect shoulder kinematics (Murgia et al., 2018; Schwartz et al., 2014). This is further evidence in support of appropriately matching control cohorts to mitigate factors that may obscure detection of changes in shoulder function by group.

In conclusion, this study compared individuals <35 and >45 years of age during 3 static poses and 2 dynamic activities, and quantified resultant kinematics of the HT, ST, and GH joints. These data provide valuable baselines for expected differences in shoulder motion by age, and detailed measurements of the ST and GH joints which were not previously reported. Data on IR of the arm behind the back were presented, highlighting areas of focus for individuals suffering from limited IR mobility. While scapular plane elevation and IR/ER in adduction ROM did not differ between groups, numerous differences in the ST and GH joints were uncovered. Protraction and tilt in the ST joint, and GH rotation and elevation, were the most likely sources of disparities between age groups and could provide targets for improving shoulder function in pathology, injury, and clinical care of the shoulder. These data highlight the importance of ST motion in overall shoulder motion, where differences up to 22° are present between younger and older individuals. These data support the need for age matching of control populations when studying shoulder (dys)function so as not to obscure variations solely based on inherent shoulder function of the reference population.

## Supplementary Material

1

## Figures and Tables

**Table 1.** Humerothoracic (HT), scapulothoracic (ST), and glenohumeral (GH) start and end orientations and ROM for scapular plane abduction and external rotation in adduction motions.

			<35 Years			>45 Years			P	P	P
			Start	End	ROM	Start	End	ROM	Start	End	ROM
**Scapular plane abduction**	**HT**	**IR/ER**	***13±6***	85±14	***72±17***	***-10±20***	84±7	***94±21***	***0.004***	0.809	***0.028***
		**Elevation**	6±6	157±12	151±15	10±4	155±6	145±5	0.102	0.712	0.266
		**Plane of elevation**	7±10	23±9	25±8	2±6	20±7	28±10	0.237	0.452	0.560

	**ST**	**Protraction**	42±9	***44±7***	7±3	47±4	***55±6***	10±6	0.136	***0.002***	0.173
		**Lateral rotation**	−14±8	32±9	47±5	−14±10	36±9	50±4	0.887	0.330	0.138
		**Tilt**	***−8±3***	***−3±4***	5±3	***−14±8***	***−10±7***	5±3	***0.041***	***0.023***	0.973

	**GH**	**IR/ER**	***40±9***	53±7	***18±6***	***23±19***	50±7	***31±16***	***0.034***	0.393	***0.029***
		**Elevation**	8±8	102±8	94±11	6±7	98±7	91±6	0.551	0.176	0.519
		**Plane of elevation**	12±7	***17±12***	21±11	12±9	***7±6***	19±7	0.979	***0.029***	0.623

**External rotation in adduction**	**HT**	**IR/ER**	−63±11	54±14	117±18	−64±8	48±9	113±8	0.803	0.323	0.521
		**Elevation**	12±5	*9±6*	7±3	15±7	*16±9*	6±3	0.415	*0.051*	0.635
		**Plane of elevation**	2±7	−1±5	7±3	8±8	−2±6	11±8	0.107	0.828	0.168

	**ST**	**Protraction**	45±8	36±9	10±5	48±5	37±8	11±8	0.380	0.903	0.626
		**Lateral rotation**	−13±8	−15±10	4±2	−13±9	−14±9	4±2	0.850	0.800	0.987
		**Tilt**	***−7±4***	*−9±4*	3±1	***−14±7***	*−15±9*	3±2	***0.030***	*0.084*	0.816

	**GH**	**IR/ER**	−16±10	93±8	109±15	−11±7	91±12	102±9	0.288	0.690	0.275
		**Elevation**	*23±8*	21±7	6±2	*30±6*	26±8	7±4	*0.053*	0.238	0.453
		**Plane of elevation**	*0±8*	3±6	7±4	*7±8*	2±13	7±6	*0.089*	0.882	0.953

Reported as mean±SD degrees where ***bold italics*** denotes significance between age groups (P≤0.050). *Italics* denote marginal significance (0.050<P≤0.100). Since substantial reorientation can occur during a dynamic motion, ROM was calculated as the maximum range (max-min) across the entirety of the performed motion, not the difference between start and end orientations. At times this resulted in the ROM exceeding the difference between start and end orientations (e.g. GH plane of elevation during scapular plane abduction).
