# Supplementary material for: Age-related differences in humerothoracic, scapulothoracic, and glenohumeral kinematics during elevation and rotation motions
Source: J Biomech. Author manuscript; Available in PMC 2022 Mar 5. (PMC7924070; doi:10.1016/j.jbiomech.2021.110266)
Supplement: 1 [file NIHMS1668034-supplement-1.docx]

**
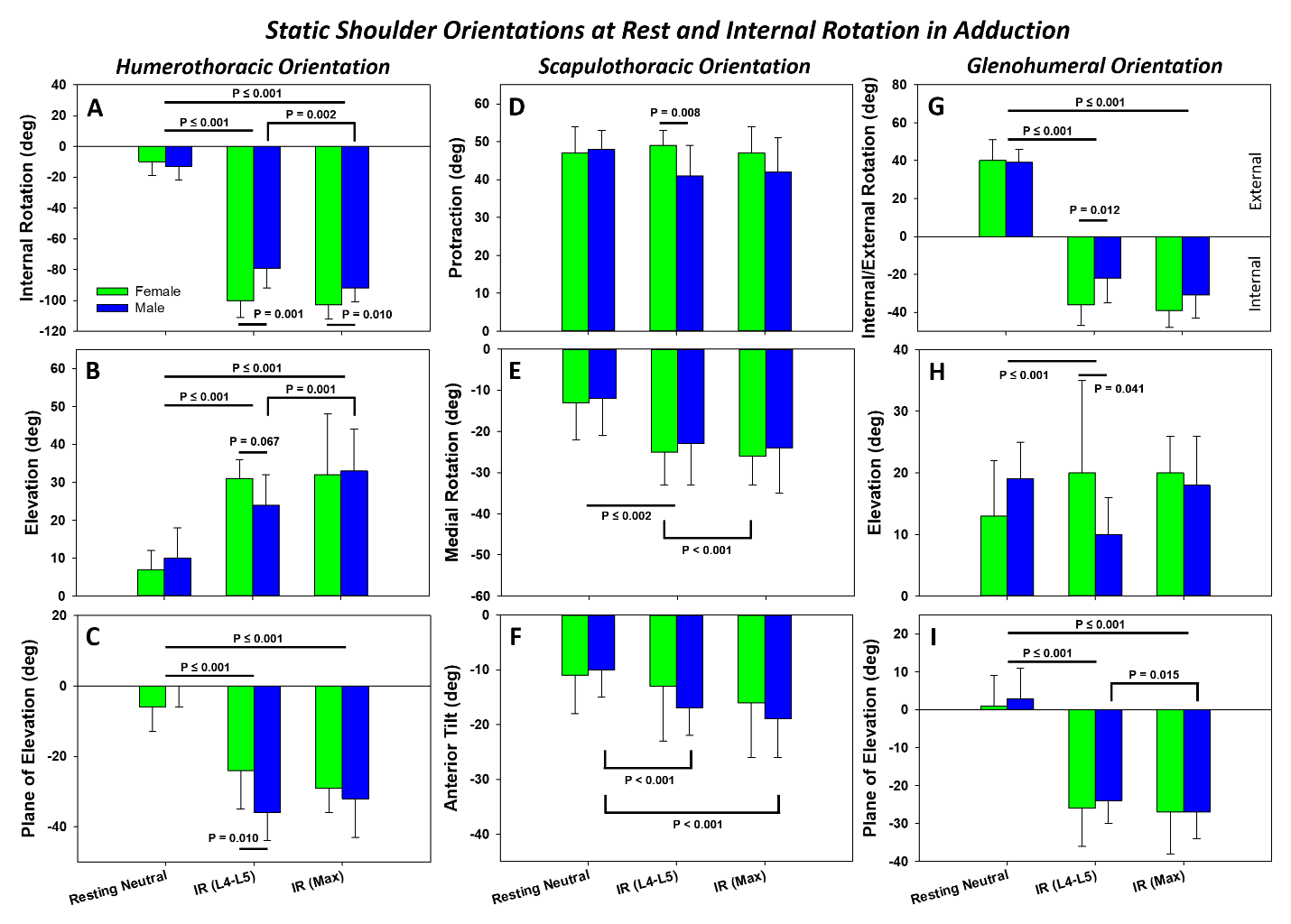
**

**Figure S1:** Humerothoracic (**A-C**), scapulothoracic (**D-F**), and glenohumeral (**G-I**) orientations during static resting neutral, internal rotation (IR) (L4-L5), and IR (max) poses, grouped by sex. Most differences in joint orientations were detected between the resting neutral and IR poses (A, B, C, E, F, G, H, I) but some differences between poses only arose within one sex (e.g. female: E; male: A, B, F, I). Sex yielded statistical differences in humerothoracic IR/ER (A) and plane of elevation (C), scapulothoracic protraction (D), and glenohumeral rotation (G) and elevation (H). Marginal significance was detected in humerothoracic elevation (B). Data are presented relative to the torso coordinate system as mean±SD.

**Table S1.** Humerothoracic (HT), scapulothoracic (ST), and glenohumeral (GH) start and end orientations and ROM for scapular plane abduction and external rotation in adduction motions.

|  |  |  | **Female** | | | **Male** | | | **P** | **P** | **P** |
| --- | --- | --- | --- | --- | --- | --- | --- | --- | --- | --- | --- |
|  |  |  | **Start** | **End** | **ROM** | **Start** | **End** | **ROM** | **Start** | **End** | **ROM** |
| **Scapular plane abduction** | **HT** | **IR/ER** | -1±22 | 85±6 | 86±22 | 4±14 | 84±14 | 80±22 | 0.570 | 0.797 | 0.539 |
|  |  | **Elevation** | 6±6 | 157±12 | 151±15 | 10±4 | 155±6 | 145±5 | 0.102 | 0.712 | 0.266 |
|  |  | **Plane of elevation** | 5±10 | 23±6 | 28±8 | 4±6 | 21±9 | 25±10 | 0.766 | 0.650 | 0.467 |
|  | **ST** | **Protraction** | *47±4* | 52±6 | 7±4 | *41±9* | 48±10 | 10±5 | *0.071* | 0.318 | 0.130 |
|  |  | **Lateral rotation** | -17±8 | 32±8 | 49±5 | -12±9 | 36±9 | 48±5 | 0.228 | 0.337 | 0.660 |
|  |  | **Tilt** | -11±7 | -6±8 | 5±4 | -11±6 | -7±6 | 5±2 | 0.885 | 0.890 | 0.903 |
|  | **GH** | **IR/ER** | 33±22 | 50±8 | 26±17 | 30±11 | 52±7 | 23±10 | 0.671 | 0.654 | 0.729 |
|  |  | **Elevation** | ***11±8*** | ***104±6*** | 93±11 | ***3±6*** | ***96±8*** | 93±7 | ***0.025*** | ***0.032*** | 0.950 |
|  |  | **Plane of elevation** | 13±10 | 12±8 | 20±10 | 12±6 | 12±13 | 20±10 | 0.821 | 0.965 | 0.920 |
| **External rotation in adduction** | **HT** | **IR/ER** | -63±11 | 54±14 | 117±18 | -64±8 | 48±9 | 113±8 | 0.803 | 0.323 | 0.521 |
|  |  | **Elevation** | 12±5 | *9±8* | 7±3 | 15±6 | *16±7* | 7±3 | 0.335 | *0.091* | 0.927 |
|  |  | **Plane of elevation** | 5±8 | -2±6 | 9±5 | 6±8 | -1±5 | 9±7 | 0.745 | 0.931 | 0.956 |
|  | **ST** | **Protraction** | 49±4 | 39±4 | 9±5 | 44±9 | 33±10 | 12±8 | 0.175 | 0.111 | 0.441 |
|  |  | **Lateral rotation** | -15±7 | -17±7 | 4±2 | -11±9 | -12±11 | 5±2 | 0.389 | 0.188 | 0.482 |
|  |  | **Tilt** | -12±7 | -14±7 | *4±2* | -9±6 | -9±7 | *2±1* | 0.486 | 0.214 | *0.073* |
|  | **GH** | **IR/ER** | -13±9 | *96±7* | 109±11 | -14±9 | *88±11* | 102±13 | 0.849 | *0.063* | 0.199 |
|  |  | **Elevation** | 27±7 | 24±7 | 7±2 | 26±9 | 23±8 | 6±4 | 0.912 | 0.939 | 0.860 |
|  |  | **Plane of elevation** | 4±9 | 5±11 | 7±6 | 2±8 | -1±8 | 6±3 | 0.598 | 0.183 | 0.688 |

Reported as mean±SD degrees where ***bold italics*** denotes significance between age groups (P≤0.050). *Italics* denote marginal significance (0.050<P≤0.100). Since substantial reorientation can occur during a dynamic motion, ROM was calculated as the maximum range (max-min) across the entirety of the performed motion, not the difference between start and end orientations. At times this resulted in the ROM exceeding the difference between start and end orientations (e.g. GH plane of elevation during scapular plane abduction).


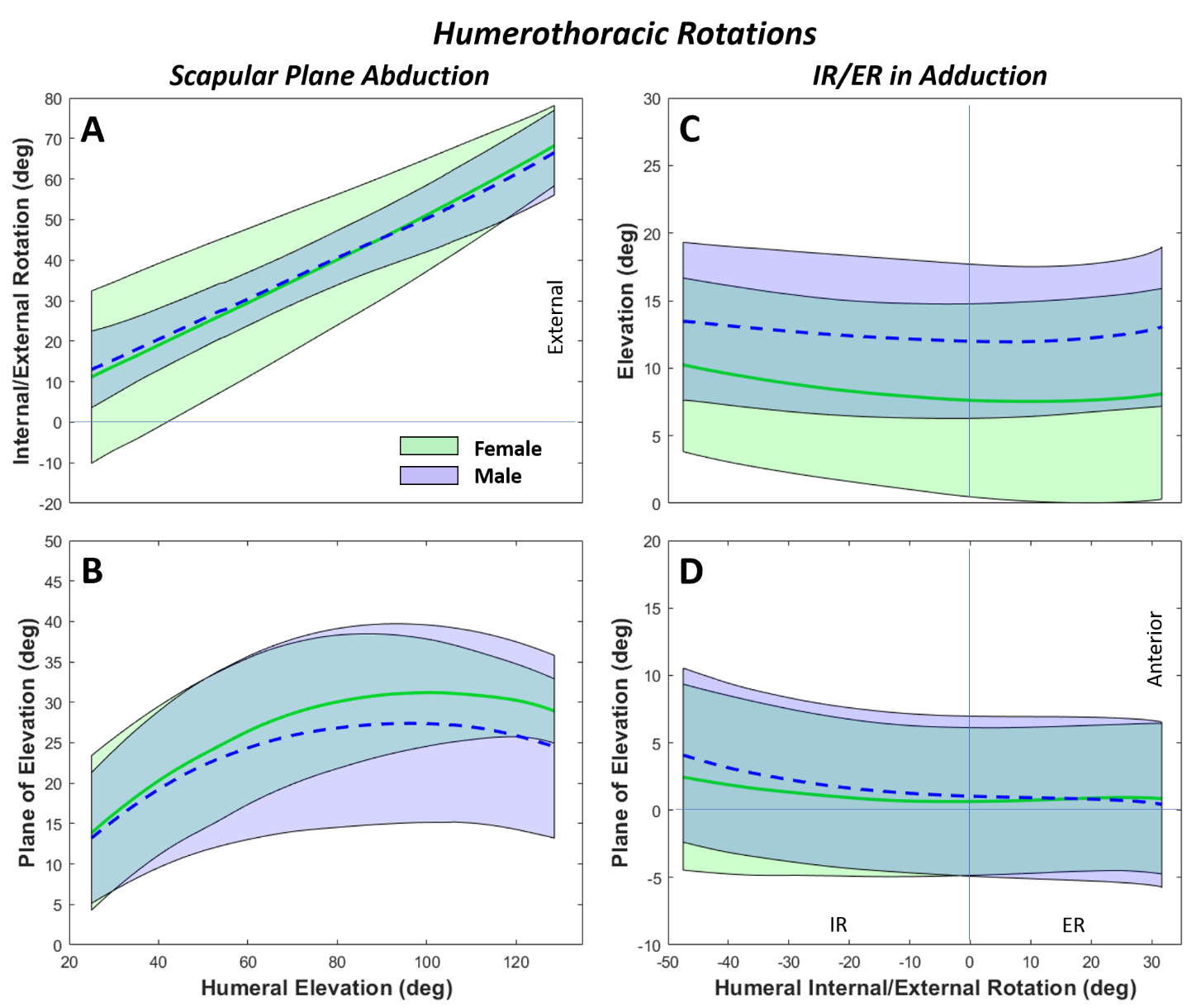


**Figure S2:** Humerothoracic rotations for scapular plane abduction (**A, B**) and internal/external rotation (IR/ER) in adduction (**C, D**). No statistical differences were detected between sexes for humerothoracic motions. Solid and dashed curves represent the mean and shaded regions represent the standard deviation of the respective populations.


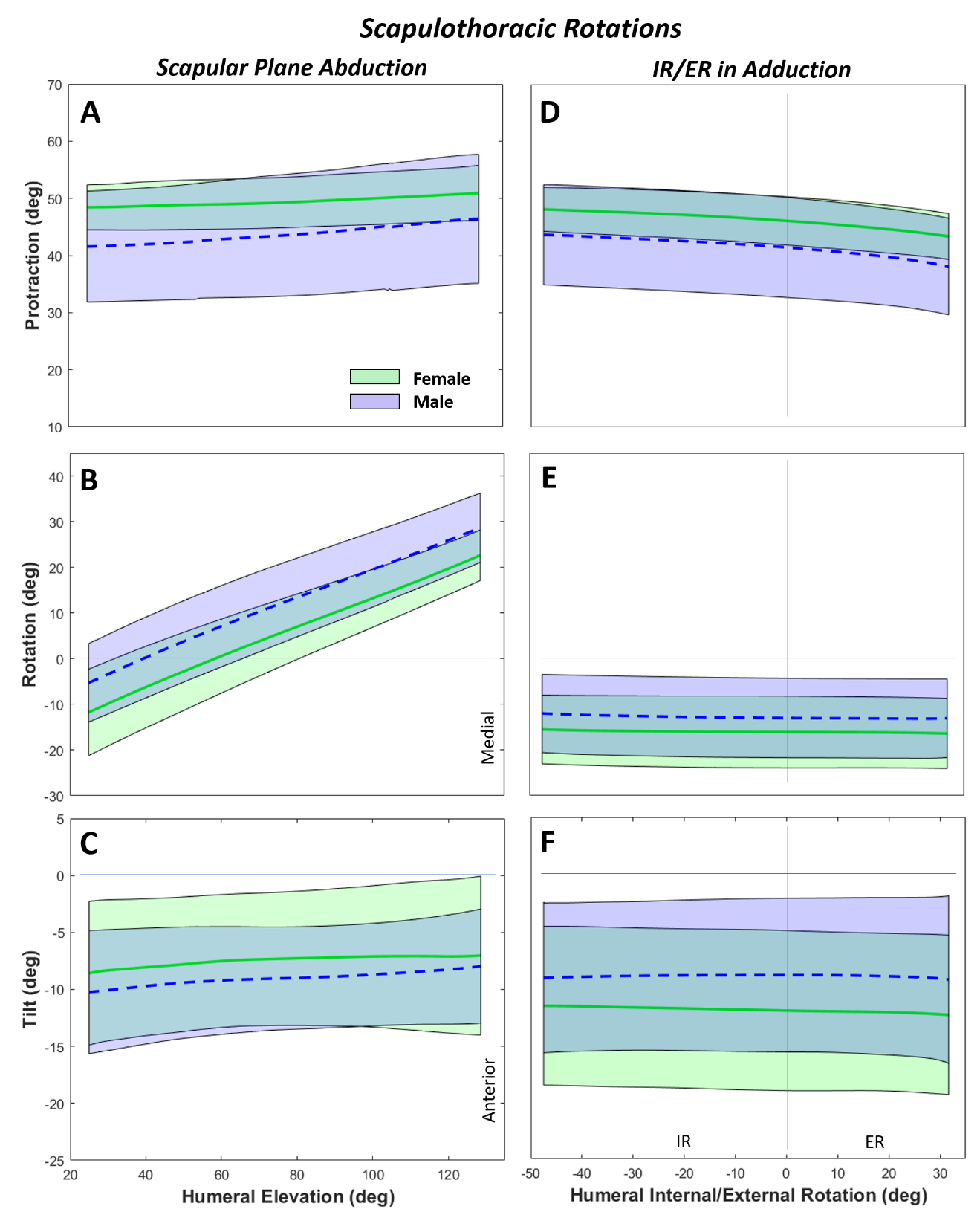


**Figure S3:** Scapulothoracic rotations for scapular plane abduction (**A-C**) and internal/external rotation (IR/ER) in adduction (**D-F**). No statistical differences were detected between sexes for scapulothoracic motions. Solid and dashed curves represent the mean and shaded regions represent the standard deviation of the respective populations.


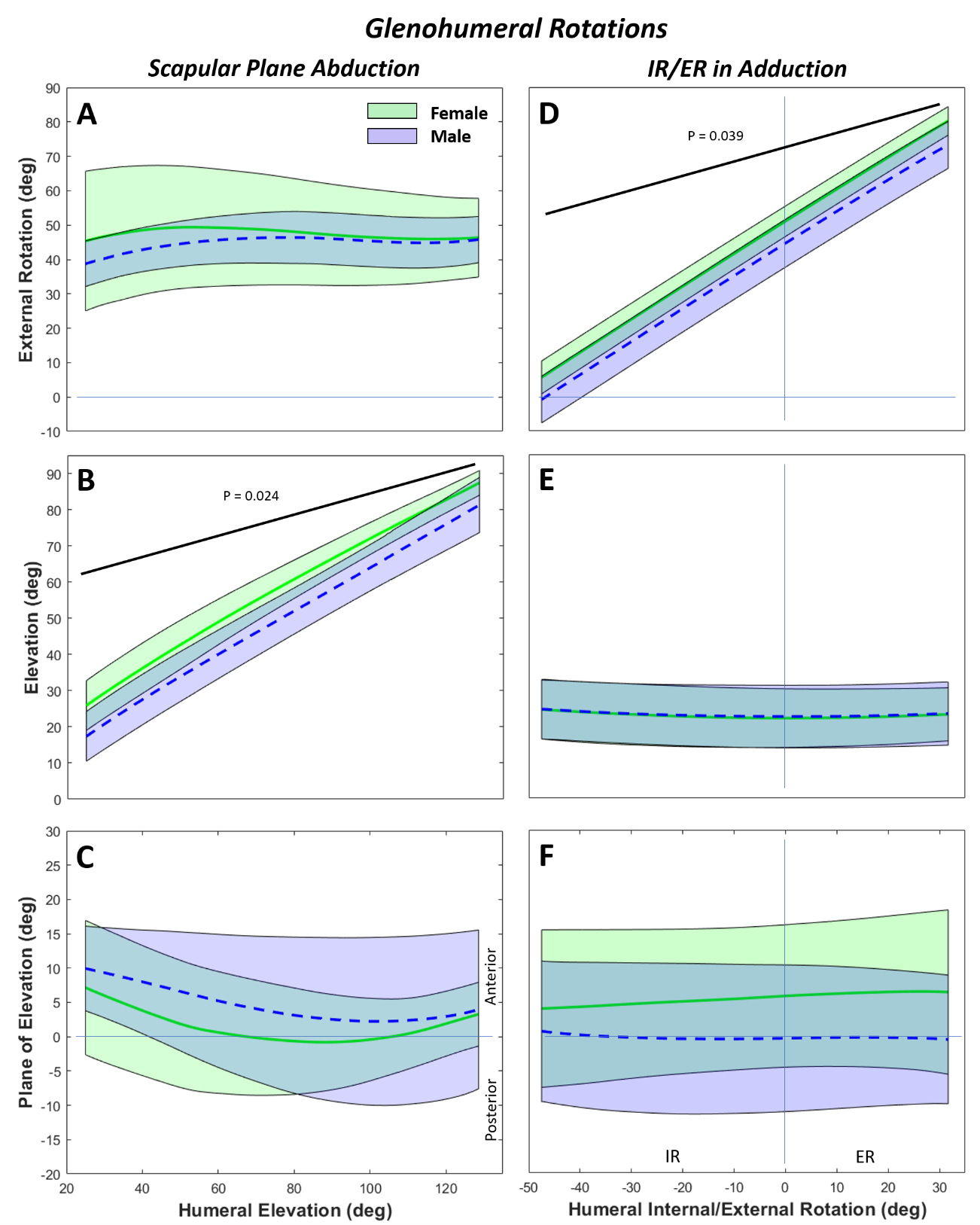


**Figure S4:** Glenohumeral rotations for scapular plane abduction (**A-C**) and internal/external rotation (IR/ER) in adduction (**D-F**). By sex, statistical differences of approximately 5-10° were observed for glenohumeral elevation during scapular plane abduction, and humeral external rotation during IR/ER in adduction. No other statistical differences were detected between sexes for glenohumeral motions. Solid and dashed curves represent the mean and shaded regions represent the standard deviation of the respective populations.


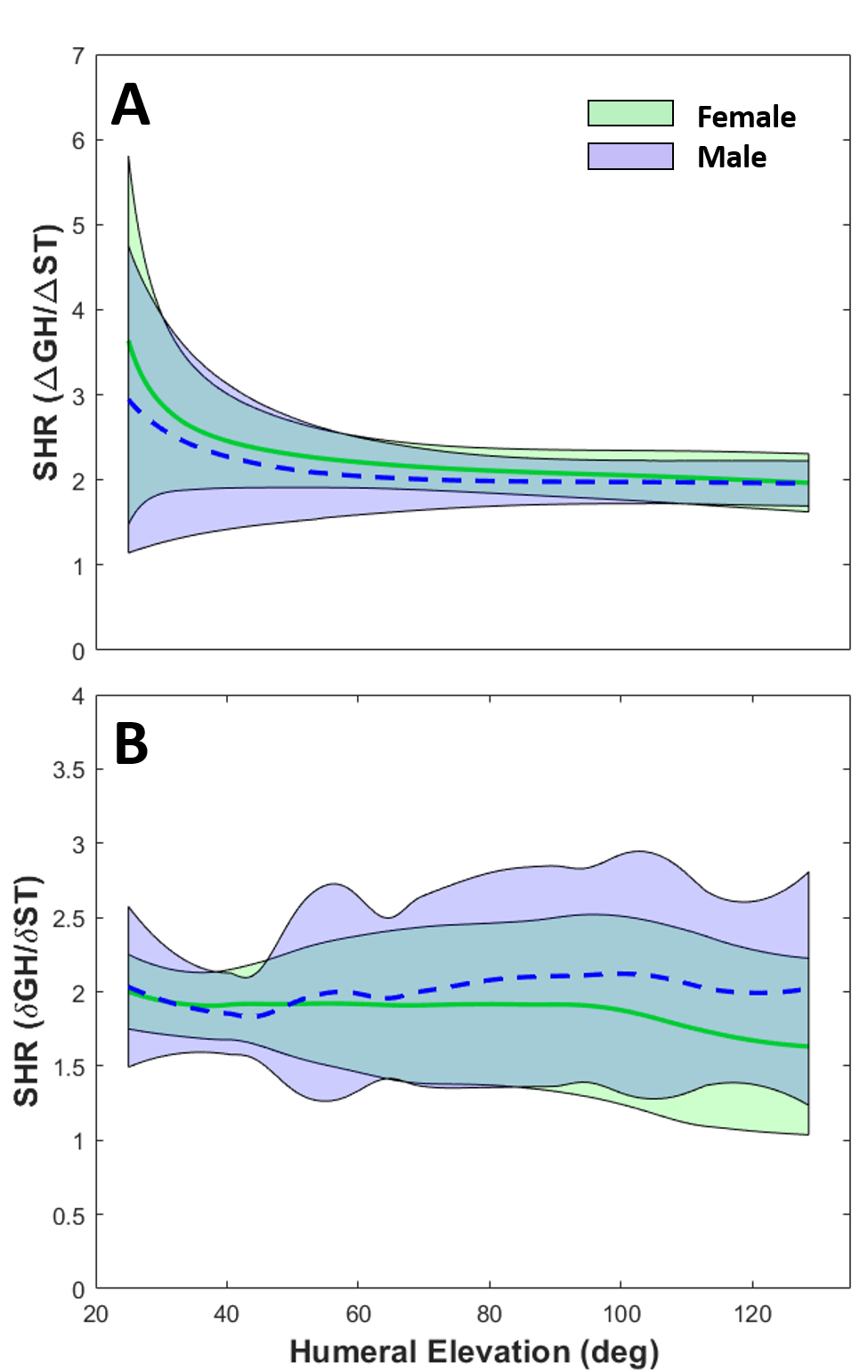


**Figure S5:** Scapulohumeral rhythm (SHR) during scapular plane abduction was calculated as the ratio of GH elevation to ST lateral rotation. **(A)** Here the SHR (Δ) represents the change relative to the initial position of the subject. Early in the elevation the SHR was dominated by glenohumeral humeral motion relative to scapulothoracic, resulting in SHR upward of 6 in some subjects, but stabilized around a 2:1 ratio above approximately 50° of humeral elevation. **(B)** Here the SHR (δ) represents the instantaneous change relative to the prior increment of elevation. A 2:1 ratio was observed across the entire range of humeral elevation. No statistical differences were detected between sexes.
